# Taxonomic Review of the Genus *Taxigramma* (Diptera, Sarcophagidae, Miltogramminae) from China †

**DOI:** 10.3390/insects16090953

**Published:** 2025-09-11

**Authors:** Zijun Liu, Yi Gai, Xiaolan Cheng, Hanqing Li, Yaqian Fan, Krzysztof Szpila, Thomas Pape, Dong Zhang

**Affiliations:** 1 School of Ecology and Nature Conservation, Beijing Forestry University, Qinghua East Road 35, Beijing 100083, China; zijun_lzj@sina.com (Z.L.); ygai9177@hotmail.com (Y.G.); lhq15865288897@163.com (H.L.); 2 Dalian International Travel Health Care Centre (Port Clinic of Dalian Customs District), Dalian 116007, China; xiaoqimengling@126.com; 3 Beijing Songshan National Nature Reserve Administration, Beijing 102100, China; fyq1018@126.com; 4 Department of Ecology and Biogeography, Faculty of Biological and Veterinary Sciences, Nicolaus Copernicus University, 87-100 Torun, Poland; szpila@umk.pl; 5 Natural History Museum of Denmark, Science Faculty, University of Copenhagen, 2100 Copenhagen, Denmark; tpape@snm.ku.dk

**Keywords:** China, COI, distribution, Miltogramminae, morphology, *Taxigramma*, taxonomy

## Abstract

The genus *Taxigramma* from China has been taxonomically reviewed. *Taxigramma pluriseta* and *T. pluton* are new to the fauna sinica, while *T. karakulensis* is removed. The male of *T. albina* is firstly recorded and described from China. An updated checklist of all eight species of *Taxigramma* known from China is presented with newly supplemented specimen examinations, biological observations and distributional information. A taxonomic key to the Chinese species of *Taxigramma* is also provided. COI sequences of *T. albina*, *T. pluriseta* and *T. pluton* are newly obtained, with a concise analysis of their genetic divergence among species of *Taxigramma*.

## Simple Summary

*Taxigramma* Perris, 1852 is a small genus within the subfamily Miltogramminae, comprising a total of 20 species. This study presents the first review of all *Taxigramma* species from China, reporting two newly recorded species, *T. pluriseta* (Pandellé, 1895) and *T. pluton* (Verves, 1984) and removing *T. karakulensis* (Enderlein, 1933), thereby raising the number of species recorded from China to eight. Based on recent collecting and examination of museum specimens, we have updated the checklist including relevant geographical and biological information, along with giving an identification key for all species of *Taxigramma* currently known from China. Additionally, we bring the first description of the male *T. albina* (Rohdendorf, 1935), and sequences of mitochondrial *cytochrome c oxidase I* (COI) are provided for *T. albina*, *T. pluriseta* and *T. pluton* for the first time, while preliminarily investigating the intra- and interspecific genetic distances of *Taxigramma* species.

## 1. Introduction

The genus *Taxigramma* Perris, 1852 is a relatively low-diversity taxon within the subfamily Miltogramminae, comprising only 20 species [1,2]. Alternative taxonomic treatments have been proposed [3,4], wherein this group is subdivided into four distinct genera (*Hilarella* Rondani, 1856, *Metopodia* Brauer & Bergenstamm, 1889, *Paragusia* Schiner, 1861 and *Taxigramma*), all of which are classified under the tribe Taxigrammini Rohdendorf, 1967 [2].

In the early taxonomic studies, only five species of this genus were reported from China: *Taxigramma elegantula* (Zetterstedt, 1844), *T. heteroneura* (Meigen, 1830), *T. multipunctata* (Rondani, 1859), *T. stictica* (Meigen, 1830) [5,6,7] and *T. karakulensis* (Enderlein, 1933) [1] [note: Xue & Chao [6] additionally reported *Hilarella siphonina* (Zetterstedt, 1844), which was subsequently treated as a junior synonym of *T. stictica*].

In subsequent studies, *Taxigramma albina* (Rohdendorf, 1935) was firstly reported from Kalamaili Mountain Ungulate Nature Reserve (hereafter referred to as Kalamaili) in China [8]. In a taxonomic revision employing Anchored Hybrid Enrichment (AHE) methodology, *Metopodia* was treated as a junior synonym of *Taxigramma* [2]. Consequently, the species previously recorded from China as *Metopodia pilicornis* (Pandellé, 1895) has been reclassified under the genus *Taxigramma*, thereby increasing the documented records of the genus *Taxigramma* from China to seven species.

Examination of specimens collected from Xinjiang Uygur Autonomous Region, China revealed two previously undocumented species, *T. pluriseta* and *T. pluton*. Aditionally, the male of *T. albina* from the Turpan–Hami Basin and Kalamaili is described for the first time. *Taxigramma karakulensis* is removed as a doubtful species in China due to the lack of detailed records and the absence of examined Chinese specimens. The Chinese *Taxigramma* fauna now includes eight species. This study successfully sequenced COI genes from seven Chinese *Taxigramma* species, including the first-reported genetic data for three species, *T. albina*, *T. pluriseta* and *T. pluton*. No specimens of *T. pilicornis* were available for destructive sampling for DNA extraction. Preliminary assessment of interspecific divergence was conducted using combined data from NCBI GenBank. We also obtained COI sequences of *T. multipunctata* from Xinjiang, Sichuan and Hainan, enabling initial analysis of its intraspecific genetic distances.

## 2. Materials and Methods

### 2.1. Collecting Sites

All specimens examined in this study were collected from China, predominantly within the Xinjiang Uygur Autonomous Region—a suitable habitat for Miltogramminae fauna. Sampling efforts focused on ecologically distinctive zones such as Turpan-Hami Basin, Karamaili and border areas.

### 2.2. Specimen Processing

Specimens of *Taxigramma* were collected by hand net, euthanized with ethyl acetate vapors and dry-pinned. Material examined in the present study are preserved in Beijing Forestry University, Beijing, China; Shenyang Normal University, Shenyang, Liaoning, China and Institute of Zoology, Chinese Academy of Sciences, Beijing, China. Dissection of terminalia followed Zhang et al. [8].

### 2.3. Morphological Study

Female terminalia and spermathecae were photographed with a Canon 750D camera (Canon, Inc., Tokyo, Japan) coupled to a ZEISS Axio Imager.D2 upright microscope (Zeiss AG., Oberkochen, Germany), while all other light microscope images were captured using the same camera mounted on an Olympus SZX16 stereomicroscope (Olympus Corp., Tokyo, Japan). Photo stacking was carried out using the software Helicon Focus 8.1.0 Pro (Helicon Soft Ltd., Kharkov, Ukraine) and then processed using Adobe Photoshop CC 2018 (Adobe Systems, Inc., San Jose, CA, USA).

Terminology follows Cumming and Wood [9]. The nomenclature and classification follow Pape [1] modified by Johnston et al. [2].

### 2.4. DNA Sequencing and Analysis

Total genomic DNA was extracted from three right legs of each individual using the QIAamp DNA Micro Kit (Qiagen GmbH., Hilden, Germany) following the manufacturer’s protocols (accession: PX133180–PX133184). A fragment near the 5′ terminus of the mitochondrial COI gene was amplified with universal primers LCO1490 and HCO2198 [10].

Electrophoretic analysis on 1% agarose gels indicated severe DNA degradation in *T. elegantula* (accession: PX133185). Consequently, amplification was performed on a mini-barcode region within the standard COI fragment, using primers miniBarcode-F and miniBarcode-R designed for degraded DNA. All primers were PAGE-purified to enhance specificity and synthesized by BGI Tech. Inc. (Shenzhen, China). Primer sequences are given in Table 1.

PCR reactions were performed in a 25-μL volume containing 12.5 μL of 2 × Taq Mastermix (Jiangsu Cowin Biotech Co., Ltd., Beijing, China), 8.5 μL of ddH_2_O, 2 μL of DNA template, and 1 μL of each primer. The thermal cycling profile included an initial denaturation at 94 °C for 5 min; followed by 35 cycles of denaturation at 94 °C for 1 min, annealing at 47 °C for 2 min, and extension at 72 °C for 1 min; with a final extension at 72 °C for 5 min. PCR products were held at 4 °C post-amplification.

Additionally, three decade-old specimens of *T. multipunctata* (accession: PX133186–PX133188) yielded no amplification using the above protocol. DNA was therefore extracted from abdominal tissue using the Hotshot method [11], followed by amplification of the mini-barcode region. PCR was conducted in a 20-μL reaction mixture containing 10 μL of 2 × Taq Mastermix, 5.2 μL of ddH_2_O, 4 μL of DNA template, and 0.4 μL of each primer. Thermocycling conditions: initial denaturation at 94 °C for 7 min 30 s; 39 cycles of 94 °C for 30 s, 55 °C for 30 s, 72 °C for 30 s; final extension at 72 °C for 7 min 30 s; followed by storage at 4 °C.

PCR products were visualized on 1% agarose gels. The unpurified PCR reactions were then directly submitted to BGI Tech. Inc.

Available COI sequences identified to species level within the genus *Taxigramma* were retrieved from NCBI GenBank (access date: 24 July 2025), yielding 11 sequences. These were combined with nine COI sequences (259–614 bp) from seven *Taxigramma* species that were generated and deposited in GenBank in this study (PX133180–PX133188) (notes see Table 2). All sequences were aligned using MUSCLE implemented in MEGA7 [12]. Pairwise genetic distances based on the Kimura 2-parameter (K2P) model [13] were calculated within MEGA7.

Neighbor-Joining (NJ) trees were reconstructed under the Kimura 2-parameter model in MEGA7. Node support was assessed with 1000 bootstrap replicates.

**Table 2:** Taxon sampling for genetic distance analysis in the present study.

Accession	Species	Collection Site	Source
KY749763	*T. multipunctata*	Israel	GenBank/Piwczyński et al. [14]
KY749761	*T. heteroneura*	Poland	GenBank/Piwczyński et al. [14]
MN411060	*T. heteroneura*	-	GenBank
KY749762	*T. hilarella*	Poland	GenBank/Piwczyński et al. [14]
MZ628314	*T. hilarella*	Finland	GenBank/Roslin et al. [15]
KY749764	*T. stictica*	Poland	GenBank/Piwczyński et al. [14]
KY749760	*T. elegantula*	Poland	GenBank/Piwczyński et al. [14]
MZ623189	*T. elegantula*	Finland	GenBank/Roslin et al. [15]
MZ623596	*T. elegantula*	Finland	GenBank/Roslin et al. [15]
MZ627095	*T. elegantula*	Finland	GenBank/Roslin et al. [15]
KY749727	*T. pilicornis*	Poland	GenBank/Piwczyński et al. [14]
PX133180	*T. albina*	Xinjiang (Yiwu)	This study
PX133181	*T. pluton*	Xinjiang (Alashankou)	This study
PX133182	*T. pluriseta*	Xinjiang (Jimsar)	This study
PX133183	*T. stictica*	Xinjiang (Huocheng)	This study
PX133184	*T. heteroneura*	Xinjiang (Alashankou)	This study
PX133185	*T. elegantula*	Xinjiang (Jeminay)	This study
PX133186	*T. multipunctata*	Xinjiang (Altay)	This study
PX133187	*T. multipunctata*	Sichuan (Panzhihua)	This study
PX133188	*T. multipunctata*	Hainan (Yinggeling)	This study

## 3. Results

### 3.1. Species Checklist

#### 3.1.1. *Taxigramma albina* (Rohdendorf, 1935) (Figure 1A,B, Figure 3A,B,F, Figure 5A,B,F,G, Figure 7A–F and Figure 10A–E)

*Hilarella albina* Rohdendorf, 1935: 114 [16].

*Taxigramma albina*: Pape, 1996: 152; Johnston et al., 2023: 143 & Suppl. fig. S4; Zhang et al., 2021: 40, 44 [1,2,8].

**Description of male:** Body length 3.80–5.50 mm.

Head. Eye bare, red. Ommatidia equally large. Fronto-orbital plate, parafacial plate and postocular strip yellow with light silvery gray pollinosity. Parafacial with one row of fine bristles, about 0.31 × as wide as fronto-orbital plate in median part. Frontal vitta yellow, with sparse pollinosity, about 0.24 × as wide as fronto-orbital plate in median part (Figure 7D). Frons at vertex about 0.34 × head width; frontal row of 8–9 bristles; outer vertical bristle differentiated from postocular bristles, two proclinate orbital bristles. Ocelli red; ocellar setae strong, directed anterolaterally. Gena ground color yellow, with black bristles and silvery gray pollinosity, height about 0.10 × eye height in lateral view (Figure 7F). Pedicel yellowish to dark yellow; first flagellomere grayish-brown, not reaching the level of vibrissal insertion, about 3.40 × as long as pedicel (Figure 7E); arista short plumose, swollen in basal 1/3 (Figure 5G). Postocular setae in 1 row. Palpus yellow, slightly expanded in distal part. Labellum brown, with fine setae. Vibrissa developed.

Thorax ground color black with silvery gray pollinosity. Chaetotaxy: acrostichals 0 + 1, dorsocentrals 2 + 3, intra-alars 0 + 1(2), postalars 2, postpronotals 2, notopleurals 2, scutellum with 1 apical, 1 lateral, 1 basal and 1 discal bristles. Pleuron with meropleurals 5, katepisternal bristles 1:1, prosternum, metasternum, proepisternal depression and postalar wall all bare.

Wing hyaline; subcostal sclerite and basicosta yellow, bare; tegula yellow, with black fine setulae; costal spine differentiated; vein R_1_ bare; node of R_2+3_ and R_4+5_ with three to five fine black setae dorsally and two or three fine black setae ventrally; cell r_4+5_ closed (Figure 5B); calypter yellowish-white, halter yellow.

Legs black except for the yellowish knee and yellowish-brown tibia and fore tarsus. Fore femur with dense and long bristles along posterodorsal, posterior, ventral and posteroventral margins, fore tibia with one median posterior bristles, fore tarsomeres 1–5 each with an elongated hair-like seta both apico-anteriorly and apico-posteriorly (Figure 3F); mid femur with two apical posterodorsal bristles, mid tibia with two or three posterodorsal bristles, and with one or two median ventral bristles; hind femur with one row of anterodorsal bristles (about 11) and one apical anteroventral bristle, hind tibia with one row of strong anterodorsal bristles (about 12), one row of anteroventral bristles (about 5) and one row of posterodorsal bristles (about 10).

Abdomen long oval, with gray pollinosity and black spots. Tergite 3 to tergite 5 each possesses one pair of lateral spots and one single median spot, with an additional pair of spots situated between the median and lateral ones. Syntergite 1 + 2 only with one pair of spots (Figure 3B). Tergite 3 and tergite 4 both with one pair of developed median marginal bristles, tergite 5 with complete row of marginal bristles.

Terminalia. Cercus and surstylus elongate, the outer margin and apex of phallus membranous (Figure 10A). Sternite 5 shield-shaped (Figure 10B).

**Redescription of female:** Body length 5.30 mm.

Body light-colored with uniformly dense white pollinosity. Head. Parafacial about 0.60 × as wide as fronto-orbital plate in median part (Figure 7A). Frontal vitta about 0.55 × as wide as fronto-orbital plate in median part. Frons at vertex about 0.40 × head width. Gena height about 0.13 × eye height in lateral view (Figure 7C). First flagellomere about 3.55 × as long as pedicel (Figure 7B).

Legs. Fore tarsus without specialized setae.

Terminalia see Figure 10C–E.

Other morphological characteristics are similar to those of the male.

**Chinese specimens examined:** Xinjiang: 1 male, Altay, Kalamaili, 1.V.2015, M. Zhang & C. Wang; 1 female, Altay, Kalamaili, 7.V.2015, M. Zhang & C. Wang; 1 male, Yiwu, Naomaohu, Yiwu Populus Forest, 20.VII.2024, Z.J. Liu; 2 males, Gaochang, Ayding Lake National Wetland Park, 7–9.VI.2025, Z.J. Liu (all BFU).

**Chinese distribution:** Xinjiang (Altay, Gaochang, Yiwu).

**Biology:** This species is psammophilous. In Kalamaili, adults were collected near water sources. It has been recorded as a kleptoparasite on multiple taxa within the superfamily Apoidea [17].

**Remarks:** This species was described based solely on three female specimens [16], while *Taxigramma* (*Hilarelliscum*) *popovi* Rohdendorf, 1935 was established exclusively from two male specimens (both nominal taxa from Uzbekistan). In Pape’s study [1], *T. popovi* was treated as a junior synonym of *T. albina*, though detailed supporting evidence remains unpublished; the taxonomic validity of these two nominal taxa requires further investigation. *Taxigramma albina* is evidently morphologically very similar to *T. hilarella* (Zetterstedt, 1844). Rohdendorf [16], who did not have males of *T. albina*, separated these two species based on small differences in proportions and setosity of the head and a generally lighter color of *T. albina*, all of which are features prone to variation. Comparing the present photos of male fore tarsus and terminalia of *T. albina* (Figure 3F and Figure 10A,B) with illustrations of the same structures for *T. hilarella* in Pape (1987, figs. 100–104) [18] indicates possible differences, of which the very slender surstylus of *T. albina* is particularly noteworthy. While the morphology is inconclusive, the molecular signal appears much stronger, with distances for *T. albina* of 5.14–5.58% to *T. hilarella* and 8.11–8.61% to *T. stictica* (Table 3).

#### 3.1.2. *Taxigramma elegantula* (Zetterstedt, 1844) (Figure 2A,B, Figure 3G, Figure 4A,B, Figure 6A,B and Figure 8A–F)

*Tachina elegantula* Zetterstedt, 1844: 1024 [19].

*Paragusia elegantula*: Verves, 2020: 17 [3].

*Taxigramma* (*Paragusia*) *elegantula*: Fan, 1992: 599; Xue & Chao, 1998: 1548 [5,6].

*Taxigramma elegantula*: Fan & Pape, 1996: 241; Pape, 1996: 152; Zhang et al., 2021: 40, 42 [1,7,8].

**Chinese specimens examined:** Neimenggu: 1 male, Erenhot, Hongor, 11.VII.1972, C.M. Chao; 3 males, Urad Rear Banner, Mt. Langshan, 29.VI.1978, C.M. Chao; Xinjiang: 1 male, 1 female, Baicheng, Akeqisu, alt. 2400 m, 17.V.1978, X.Z. Zhang; 1 male, 1 female, Akto, Aketashi, alt. 2400 m, 27.VI.1987, X.Z. Zhang; 4 males, 1 female, Taxkorgan, Mazha, alt. 3900 m, 3.VII.1987, X.Z. Zhang; 8 males, 2 females, Akto, Qiakelike, alt. 3250 m, 13.VII.1987, X.Z. Zhang; 1 female, Yecheng, Akeqige, alt. 2700 m, 13.VIII.1987, X.Z. Zhang; 1 male, Yecheng, Supikeya, alt. 2700–2850 m, 17.VIII.1987, X.Z. Zhang; 1 female, Qiemo, Aqiang, alt. 3000 m, 24.VII.1988, X.Z. Zhang; 1 female, Akto, Wuyitake, alt. 2650 m, 8.VIII.1989, X.Z. Zhang; 2 females, Taxkorgan, Laqigu, alt. 3550 m, 16.VIII.1989, X.Z. Zhang (all IZCAS); Xinjiang: 2 female, Shule, alt. 1280 m, 10.V.2007, M.L. Sheng (both SYNU); Ningxia: 4 males, 2 females, Lingwu, 4–29.VI.2012; Xinjiang: 20 males, 6 females, Altay, Kalamaili, 9.V–9.VI.2015, M. Zhang; 34 males, 11 females, Altay, Kalamaili, 26.IV–4.VII.2015, M. Zhang & C. Wang; 1 male, Altay, Kalamaili, 12.V.2024, Z.J. Liu; 1 male, 1 female, Jeminay, Jeminay Town, 7.VI.2024, Z.J. Liu; 1 female, Barkol, Xiheigou, 12.VIII.2025, Z.J. Liu; 5 males, 1 female, Yiwu, Rezibulake, 16–17.VIII.2025, Z.J. Liu; 1 female, Yizhou, Baishanquan Service Area on G7 Beijing-Xinjiang Expressway (towards Beijing), 17.VIII.2025, Z.J. Liu; 1 male, Ruoqiang, Lop Nur Wild Camel National Nature Reserve, 26.VIII.2025, Y. Gai (all BFU).

**Chinese distribution:** Neimenggu (Erenhot, Urad Rear Banner), Ningxia (Lingwu), Xinjiang (Akto, Altay, Baicheng, Barkol, Jeminay, Qiemo, Ruoqiang, Shule, Taxkorgan, Yecheng, Yiwu, Yizhou).

**Biology:** In Kalamaili, adults can be abundantly collected near water sources in May. This species has been recorded as a kleptoparasite with hosts across Apoidea and Formicoidea [17].

#### 3.1.3. *Taxigramma heteroneura* (Meigen, 1830) (Figure 1C, Figure 3C,J, Figure 5C and Figure 7G–I)

*Miltogramma heteroneura* Meigen, 1830: 367 [20].

*Taxigramma heteroneura*: Fan, 1992: 599; Fan & Pape, 1996: 243; Pape, 1996: 153; Verves, 2020: 17; Xue & Chao, 1998: 1548 [1,3,5,6,7].

**Chinese specimens examined**: Neimenggu: 3 males, Shangdu, 19.VI.1971; Hebei: Weichang, Saihanba, alt. 1500 m, 22.VII.1985, X.Z. Zhang; Xinjiang: 1 male, Zhaosu, alt. 1620 m, 7.VIII.1957, G. Wang (all IZCAS); Neimenggu: 25 males, Qahar Right Rear Banner, Volcano No. 6 of the Ulan Had Volcanic Cluster, 20.VIII.2025, Z.J. Liu; Xinjiang: 1 male, Altay, Kalamaili, 9.VI.2014, M. Zhang; 4 males, Alashankou, 28.V.2024, Z.J. Liu; 1 male, Yumin, 5.VI.2024, Z.J. Liu; 1 male, Barkol, Barkol Grassland, 24.VI.2024, Z.J. Liu; 1 male, Barkol, Santanghu, 13–15.VIII.2025, Z.J. Liu; 1 male, Yiwu, Rezibulake, 16–17.VIII.2025, Z.J. Liu (all BFU).

**Chinese distribution:** Neimenggu (Qahar Right Rear Banner, Shangdu), Hebei (Weichang), Xinjiang (Alashankou, Altay, Barkol, Yiwu, Yumin, Zhaosu).

**Biology.** In Alashankou, Barkol and Yumin, adults occur in sparsely vegetated grasslands, where individuals perch or hover near the entrances of ground-nesting bee burrows, while in Kalamaili, it can be found on sun-exposed earthen slopes near water sources. This species has been recorded as a kleptoparasite of solitary bees (Hymenoptera: Apoidea) [17].

#### 3.1.4. *Taxigramma multipunctata* (Rondani, 1859) (Figure 2C,D, Figure 4C,D, Figure 6C,D and Figure 8G–L)

*Heteropterina multipunctata* Rondani, 1859: 211 [21].

*Paragusia multipunctata*: Fan & Pape, 1996: 241; Verves, 2020: 17 [3,7].

*Taxigramma multipunctata*: Pape, 1996: 154; Zhang et al., 2021: 40, 43 [1,8].

*Taxigramma* (*Eutaxigramma*) *multipunctata*: Fan, 1992: 599; Xue & Chao, 1998: 1548 [5,6].

**Chinese specimens examined:** Heilongjiang: 1 female, Tailai, 25.VI.1970; Neimenggu: 1 male, Erenhot, 31.VII.1972; Xinjiang: 1 female, Jimsar, 17.V.1955, S.J. Ma, K.L. Xia & Y.L. Chen; Sichuan: 1 female, Panzhihua, alt. 1200 m, 17.IV.1986; Yunnan: 1 female, Lancang, alt. 1000 m, 2.VIII.1957, L.C. Zang; 1 female, Xishuangbanna, Mengzhe, alt. 1200 m, 19.VI.1958, Z.Z. Chen (all IZCAS); Xinjiang: 4 males, 1 female, Altay, Kalamaili, 11–16.VIII.2009, D. Zhang; 18 males, 21 females, Altay, Kalamaili, 7.V–16.VI.2014, M. Zhang; 11 males, 2 females, Altay, Kalamaili, 26.IV–9.VII.2015, M. Zhang; 1 male, Alashankou, 27.V.2024, Z.J. Liu; 2 males, 2 females, Yizhou, Baishanquan Service Area on G7 Beijing-Xinjiang Expressway (towards Ürümqi), 17.VIII.2025, Z.J. Liu; 5 males, 2 females, Yizhou, Miaoergou, 5.VIII.2025, Z.J. Liu; 3 males, Yiwu, Rezibulake, 16–17.VIII.2025, Z.J. Liu; Hainan: 2 males, Yinggeling, 28.VI.2016, L.P. Yan & C. Wang; Sichuan: 4 males, Panzhihua, 10–11.VII.2015, L.P. Yan & C. Wang (all BFU).

**Chinese distribution:** Heilongjiang (Tailai), Neimenggu (Erenhot), Xinjiang (Alashankou, Altay, Jimsar, Yiwu, Yizhou), Hainan (Yinggeling); Sichuan (Panzhihua), Yunnan (Lancang, Xishuangbanna).

**Biology:** In Kalamaili, adults can be abundantly collected near water sources during May to June. This species has been recorded as a kleptoparasite of solitary bees (Hymenoptera: Apoidea) [17].

#### 3.1.5. *Taxigramma pilicornis* (Pandellé, 1895) (Figure 1E,F, Figure 3E, Figure 5E,H and Figure 7M–O)

*Metopia pilicornis* Pandellé, 1895: 304 [22].

*Metopodia pilicornis*: Fan & Pape, 1996: 240; Pape, 1996: 104; Verves, 2020: 12 [1,3,7].

*Metopodia grisea* [misidentification: not *Miltogramma grisea* Meigen, 1824]: Fan, 1992: 596, 597; Xue & Chao, 1998: 1543, 1544 [5,6].

*Taxigramma pilicornis*: Johnston et al., 2023: 149 [2].

**Chinese specimens examined:** Beijing: 1 male, VII.1933; 3 males, Haidian, Wofo Temple, 25.V.1961; 1 male, Yanqing, Badaling, alt. 700 m, 28.VI.1962, S.Y. Wang; 1 male, Yanqing, 26.VI.1970 (all IZCAS).

**Chinese distribution:** Beijing (Haidian, Yanqing).

**Biology:** The species is frequently observed in sandy habitats along riverbanks and coastal shorelines [23,24]. *Sphex funerarius* Gussakovskij, 1934 (Hymenoptera: Sphecidae) is the only currently known host for this species [25].

#### 3.1.6. *Taxigramma pluriseta* (Pandellé, 1895) (Figure 2E,F, Figure 3H, Figure 4E,F, Figure 5J, Figure 6E,F and Figure 9A–F)

*Heteropterina pluriseta* Pandellé, 1895: 313 [22].

*Taxigramma pluriseta*: Pape, 1996: 154 [1].

**Redescription of male:** Body length 3.80–5.00 mm.

Head. Eye bare. Ommatidia equally large. Fronto-orbital plate, parafacial plate and postocular strip yellow with light silvery gray pollinosity. Parafacial with one or two rows of fine bristles, about 0.56 × as wide as fronto-orbital plate in median part. Frontal vitta yellow, with sparse pollinosity, about 0.56 × as wide as fronto-orbital plate in median part (Figure 9A). Frons at vertex about 0.37 × head width; frontal row of 6–7 bristles; outer vertical bristle differentiated from postocular bristles, two proclinate orbital bristles. Ocelli red; ocellar setae strong, directed anterolaterally. Gena ground color yellow, with black bristles and silvery gray pollinosity, height about 0.16 × eye height in lateral view (Figure 9C). Pedicel yellowish-white; first flagellomere yellowish-white to greyish-white, not reaching the level of vibrissal insertion, about 2.45 × as long as pedicel (Figure 9B); arista almost bare, only pubescent, swollen in basal 1/3 (Figure 5J). Postocular setae in 1 row. Palpus yellow, slightly expanded in distal part. Labellum brown, with fine setae. Vibrissa developed.

Thorax ground color black with silvery gray pollinosity. Chaetotaxy: acrostichals 1(2) + 1, dorsocentrals 2 + 3, intra-alars 1 + 2(3), postalars 2, postpronotals 2, notopleurals 2, scutellum with 1 apical, 1 lateral, 1 basal and 1 discal bristles. Pleuron with meropleurals 2–4, katepisternal bristles 1:1, prosternum, metasternum, proepisternal depression and postalar wall all bare.

Wing hyaline; subcostal sclerite and basicosta yellow, bare; tegula yellow, with black fine setulae; costal spine differentiated; vein R_1_ bare; node of R_2+3_ and R_4+5_ with one or two fine black setae dorsally and one fine black seta ventrally or absent; cell r_4+5_ distinctly petiolate (Figure 6E); calypter yellowish-white, halter yellow.

Legs mostly black except for the yellowish knee. Fore femur with dense and long bristles along posterodorsal, posterior, ventral and posteroventral margins, fore tibia with one submedian posterior bristles, fore tarsus with about eight short fine setae (Figure 3H); mid femur with two apical posterodorsal bristles, mid tibia with two to four posterodorsal bristles, and with one or two median ventral bristles; hind femur with one row of anterodorsal bristles (about 9) and one apical anteroventral bristle, hind tibia with one row of strong anterodorsal bristles (about 5), three or four anteroventral bristles and three to six posterodorsal bristles.

Abdomen long oval, with gray pollinosity and black spots. Syntergite 1 + 2 to tergite 5 each possesses one pair of lateral spots and one single median spot, with an additional pair of spots situated between the median and lateral ones, with those lateral and median spots on the syntergite 1 + 2 being indistinct or absent, while the median spot and adjacent spots on the tergite 5 sometimes are fused (Figure 4E). Syntergite 1 + 2 only with one pair of fine median marginal bristles or absent. Tergite 3 and tergite 4 both with one pair of developed median marginal bristles, tergite 5 with complete row of marginal bristles.

**Redescription of female:** Body length 3.50–4.80 mm.

Head. Parafacial about 0.66 × as wide as fronto-orbital plate in median part (Figure 9D). Frontal vitta about 0.65 × as wide as fronto-orbital plate in median part. Frons at vertex about 0.41 × head width. Gena height about 0.17 × eye height in lateral view (Figure 9F). First flagellomere about 1.75 × as long as pedicel (Figure 9E).

Legs. Fore tarsus without specialized setae.

Other morphological characteristics are similar to those of the male.

**Chinese specimens examined:** Xinjiang: 2 males, Shawan, alt. 415 m, 13.VI.1957, C.P. Hong; 1 female, Manas, alt. 400–550 m, 4.VII.1957, G. Wang (all IZCAS); Xinjiang: 9 males, 2 female, Altay, Kalamaili, 9–16.VI.2014, M. Zhang; 1 male, Jimsar, Przewalski’s Horse Breeding and Research Center, 14.V.2024, Z.J. Liu (all BFU).

**Chinese distribution:** Xinjiang (Altay, Jimsar, Manas, Shawan).

**Biology:** In Kalamaili and Jimsar, adults can be found near water sources or visiting flowering plants. This species has only been recorded as a kleptoparasite on *Prionyx kirbii* (Vander Linden, 1827) (Hymenoptera: Sphecidae) [17].

#### 3.1.7. *Taxigramma pluton* (Verves, 1984) (Figure 1D, Figure 3D, Figure 5D,K and Figure 7J–L)

*Paragusia pluton* Verves, 1984: 544 [26].

*Taxigramma pluton*: Pape, 1996: 154 [1].

**Redescription of male:** Body length 4.50–5.50 mm.

Head. Eye bare. Ommatidia equally large. Fronto-orbital plate, parafacial plate and postocular strip brown with silvery gray pollinosity. Parafacial about 0.53 × as wide as fronto-orbital plate in median part, bears two rows of fine bristles, with one complete and another one restricted to the upper part. Frontal vitta with sparse pollinosity, about 0.48 × as wide as fronto-orbital plate in median part (Figure 7J). Frons at vertex about 0.35 × head width; frontal row of 9–10 bristles; outer vertical bristle differentiated from postocular bristles, two or three proclinate orbital bristles. Ocelli red; ocellar setae strong, directed anterolaterally. Gena ground color brown, with black bristles and silvery gray pollinosity, height about 0.14 × eye height in lateral view (Figure 7L). Pedicel yellow in apical part; first flagellomere grayish-brown, not reaching the level of vibrissal insertion, about 3.00 × as long as pedicel (Figure 7K); arista almost bare, only pubescent, swollen in basal 1/3 (Figure 5K). Postocular setae in 2 rows. Palpus yellow, slightly expanded in distal part. Labellum brown, with fine setae. Vibrissa developed.

Thorax ground color black with gray pollinosity. Chaetotaxy: acrostichals 1(2) + 1(2), dorsocentrals 2 + 3, intra–alars 0(1) + 2, postalars 2, postpronotals 2, notopleurals 2, scutellum with 1 apical, 1 lateral, 1 basal and 1 discal bristles. Pleuron with meropleurals 3–4, katepisternal bristles 1:1, prosternum, metasternum, proepisternal depression and postalar wall all bare.

Wing hyaline; subcostal sclerite and basicosta yellow, bare; tegula yellow, with black fine setulae; costal spine differentiated; vein R_1_ bare; node of R_2+3_ and R_4+5_ with one to five fine black setae dorsally and one or two fine black setae ventrally; cell r_4+5_ closed (Figure 5D); calypter yellowish-white, halter brown.

Legs mostly black except for the yellowish knee. Fore femur with dense and long bristles along posterodorsal, posterior, ventral and posteroventral margins, fore tibia with one row of slender anterodorsal bristles (about 5), one row of slender posterodorsal bristles (about 6) and one submedian posterior bristles; mid femur with two apical posterodorsal bristles, mid tibia with one row of posterodorsal bristles (about 6), and with one or two median ventral bristles; hind femur with one row of anterodorsal bristles (about 9) and one apical anteroventral bristle, hind tibia with one row of strong anterodorsal bristles (about 7), three or four anteroventral bristles and five to seven posterodorsal bristles.

Abdomen long oval, with gray pollinosity and black spots. Syntergite 1 + 2 to tergite 5 each possess one pair of lateral spots, with those on the syntergite 1 + 2 being indistinct or absent. Tergite 3 to tergite 5 each with indistinct median spot, typically manifests as two small spots at the base of the median marginal bristles, exhibiting a indistinct medial discontinuity. Syntergite 1 + 2 to tergite 5 each with an additional pair of indistinct spots situated between the median and lateral ones, while the median spot and adjacent spots on the tergite 5 sometimes are fused (Figure 3D). Tergite 3 and tergite 4 both with one pair of developed median marginal bristles, tergite 5 with complete row of marginal bristles.

**Chinese specimens examined:** Xinjiang: 1 male, Akto, Aketashi, alt. 2400 m, 27.VI.1987, X.Z. Zhang (IZCAS); Xinjiang: 1 male, Qinghe, Bulgan River Beaver Nature Reserve, 8.VIII.2015, Y.Q. Ge; 2 males, Alashankou, 28.V.2024, Z.J. Liu; 12 males, Yizhou, Tianshengquan Village, 7–8.VIII.2025, Z.J. Liu; 1 male, Yizhou, Shiyaofang, 8.VIII.2025, Z.J. Liu; 1 male, Barkol, Xiheigou, 12.VIII.2025, Z.J. Liu; 10 males, Yiwu, Rezibulake, 16–17.VIII.2025, Z.J. Liu (all BFU).

**Chinese distribution:** Xinjiang (Akto, Alashankou, Barkol, Qinghe, Yiwu, Yizhou).

**Biology:** In Alashankou, adults occur in sparsely vegetated grasslands.

#### 3.1.8. *Taxigramma stictica* (Meigen, 1830) (Figure 2G,H, Figure 3I, Figure 4G,H, Figure 5I, Figure 6G,H and Figure 9G–L)

*Miltogramma stictica* Meigen, 1830: 367 [20].

*Miltogramma siphonina* Zetterstedt, 1844: 1213 [19].

*Taxigramma stictica*: Pape, 1996: 154 [1].

*Hilarella siphonina*: Xue & Chao, 1998: 1536, 1537 [6].

*Hilarella stictica*: Fan, 1996: 598; Fan & Pape, 1996: 239; Verves, 2020: 17; Xue & Chao, 1998: 1537 [3,5,6,7].

**Chinese specimens examined:** Neimenggu: 2 males, 1 female, Qahar Right Rear Banner, Temurtei, 4–8.VI.1971; 3 males, 3 females, Qahar Right Rear Banner, Temurtei, 25.VII–1.VIII.1971; 11 males, 4 females, Qahar Right Rear Banner, Temurtei, 12–30.VIII.1971; 2 males, Erenhot, 23.VIII. 1971; 1 female, Xilinhot, 19.VIII.1972; Hebei: 1 male, 1 female, Weichang, Saihanba, alt. 1500 m, 22.VII.1985, X.Z. Zhang (all IZCAS); Neimenggu: 1 female, Tumed Left Banner, Shaerqin, 14–28.IX (SYNU); Neimenggu: 2 males, 1 female, Qahar Right Rear Banner, Volcano No. 6 of the Ulan Had Volcanic Cluster, 20.VIII.2025, Z.J. Liu; Beijing: 1 male, 1 female, Yanqing, Mt. Songshan, 1–5.VI.2009; 1 male, Mentougou, Xiaolongmen, 6–12.VII.2009, R. Bi & F. Li; Shanxi: 1 female, Lingqiu, Xiaguan, 11.VI.1980, M.F. Wang; 1 female, Shanyin, Shangliyangquan, 12.VII.1980, M.F. Wang; Xinjiang: 4 males, Huocheng, Guozigou, 30.V.2024, Z.J. Liu; 9 males, 1 female, Barkol, Barkol Grassland, 24.VI.2024, Z.J. Liu; 1 male, Barkol, Barkol Grassland, 11.VIII.2025, Z.J. Liu; 2 males, 1 female, Yiwu, Rezibulake, 16–17.VIII.2025, Z.J. Liu (all BFU).

**Chinese distribution:** Neimenggu (Qahar Right Rear Banner, Erenhot, Tumed Left Banner, Xilinhot), Beijing (Mentougou, Yanqing), Hebei (Weichang), Shanxi (Lingqiu, Shanyin), Xinjiang (Barkol, Huocheng, Yiwu).

**Biology:** In Barkol and Huocheng, adults occur in sparsely vegetated grasslands, where individuals perch or hover near the entrances of ground-nesting bee burrows. The known hosts of this kleptoparasitic species include multiple species within the superfamily Apoidea [17].

### 3.2. Key to Known Chinese Species of Genus Taxigramma

**Figure 1:**
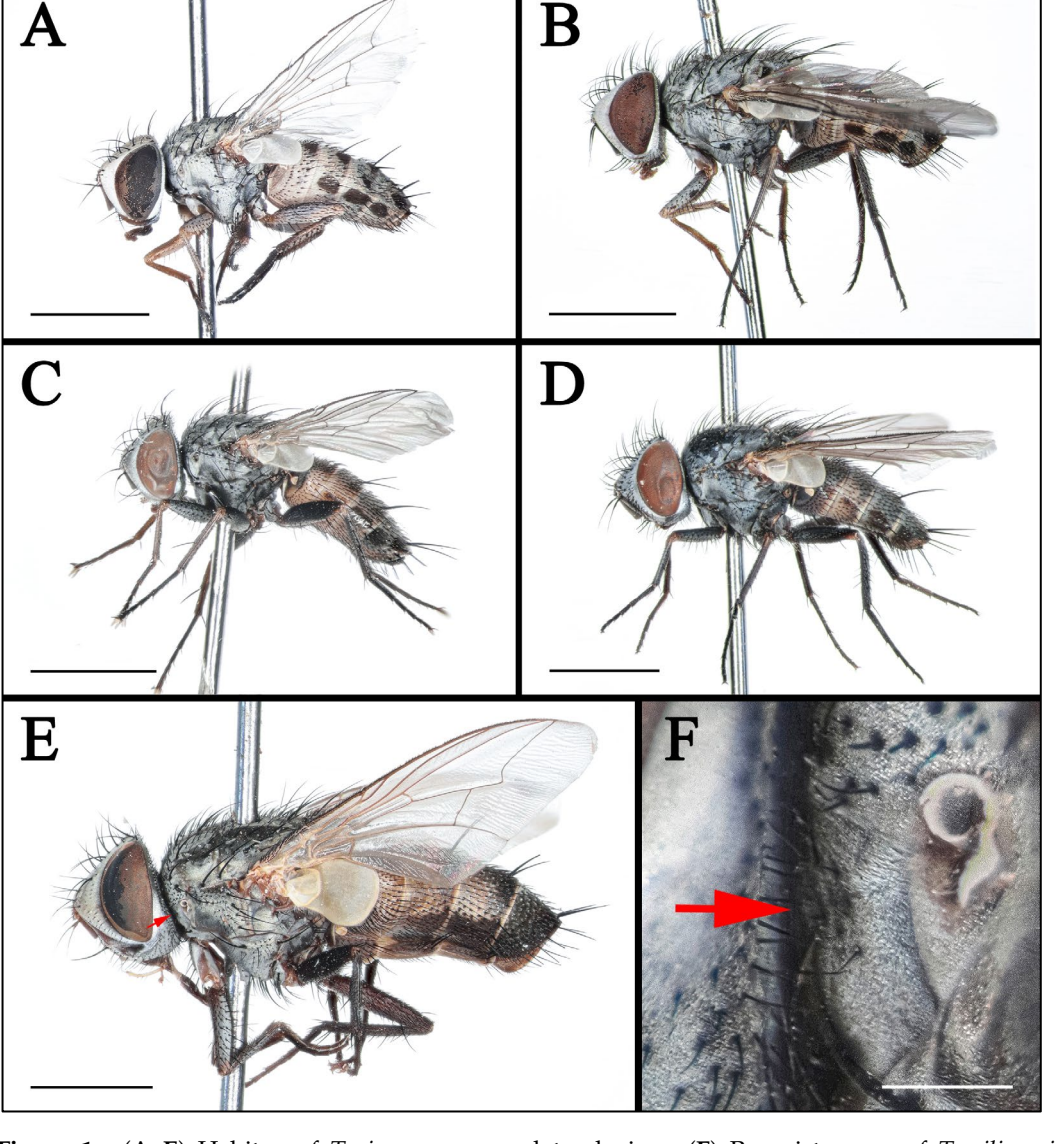
(**A**–**E**) Habitus of *Taxigramma* spp., lateral view; (**F**) Proepisternum of *T. pilicornis*. (**A**) *T. albina*, female; (**B**) *T. albina*, male; (**C**) *T. heteroneura*, male; (**D**) *T. pluton*, male; (**E**,**F**) *T. pilicornis*, male; arrow showing proepisternal depression setose. Scale bars: (**A**–**E**) 2 mm; (**F**) 0.2 mm.

**Figure 2:**
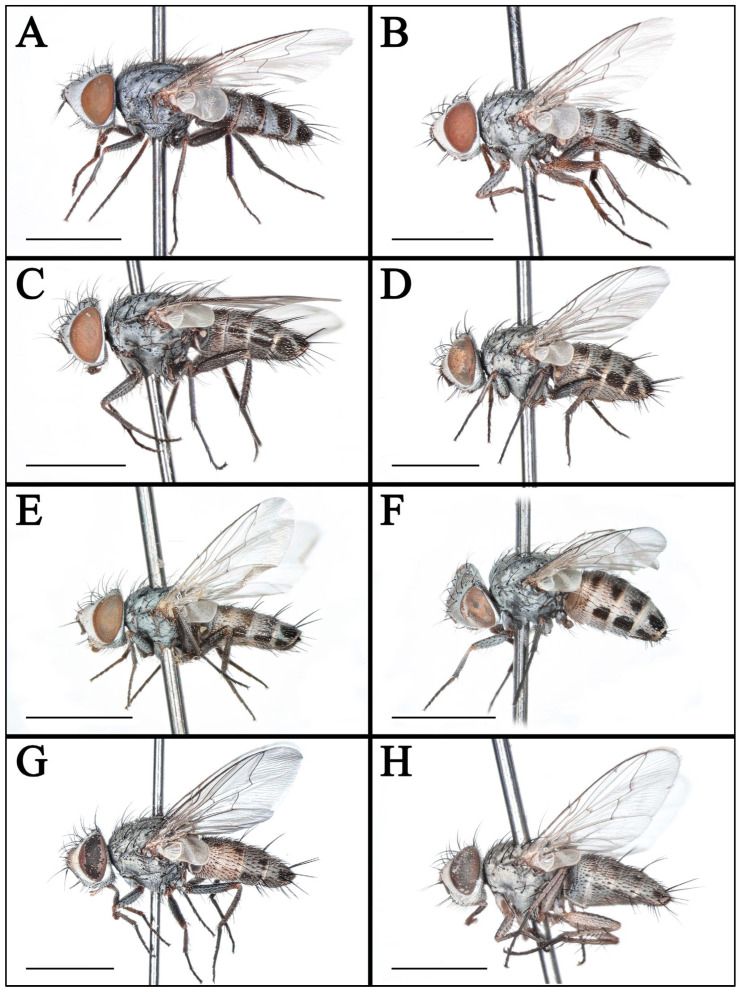
Habitus of *Taxigramma* spp., lateral view. (**A**) *T. elegantula*, male; (**B**) *T. elegantula*, female; (**C**) *T. multipunctata*, male; (**D**) *T. multipunctata*, female; (**E**) *T. pluriseta*, male; (**F**) *T. pluriseta*, female; (**G**) *T. stictica*, male; (**H**) *T. stictica*, female. Scale bars: (**A**–**H**) 2 mm.

**Figure 3:**
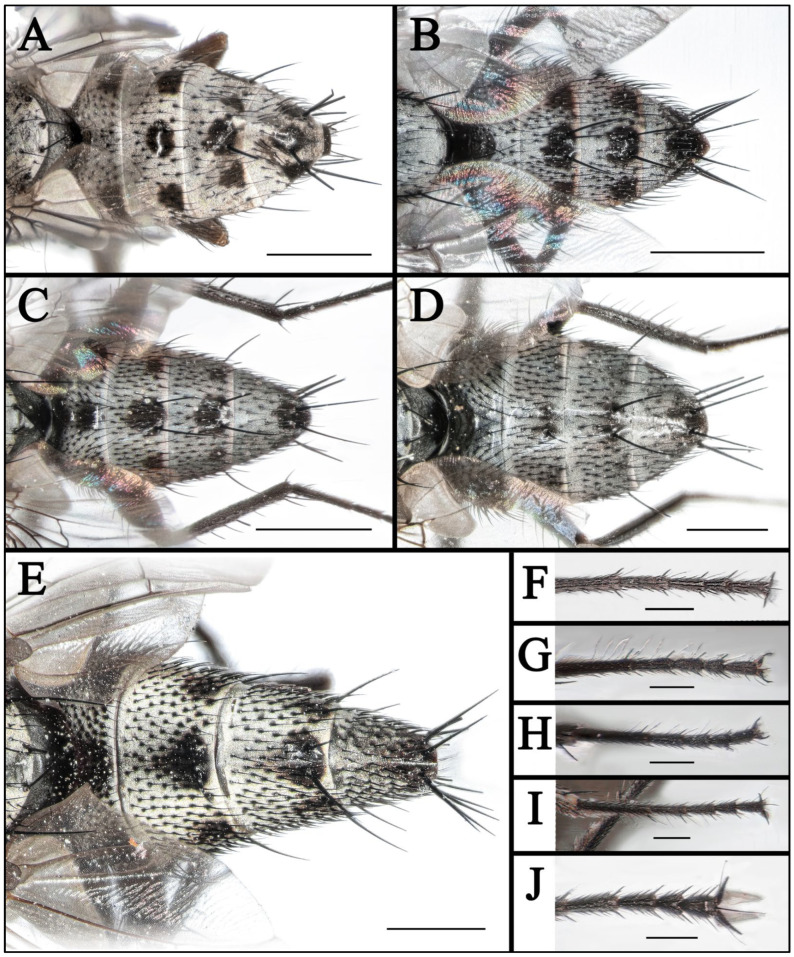
(**A**–**E**) Abdomina of *Taxigramma* spp., dorsal view; (**F**–**H**) fore tarsi of male *Taxigramma* spp., dorsal view; (**I**) claw of male *T. heteroneura*, dorsal view. (**A**) *T. albina*, female; (**B**) *T. albina*, male; (**C**) *T. heteroneura*, male; (**D**) *T. pluton*, male; (**E**) *T. pilicornis*, male; (**F**) *T. albina*, male, right fore leg; (**G**) *T. elegantula*, male, right fore leg; (**H**) *T. pluriseta*, male, right fore leg; (**I**) *T. stictica*, male, left fore leg; (**J**) *T. heteroneura*, male, right fore leg. Scale bars: (**A**–**E**) 1 mm; (**F**–**J**) 0.2 mm.

**Figure 4:**
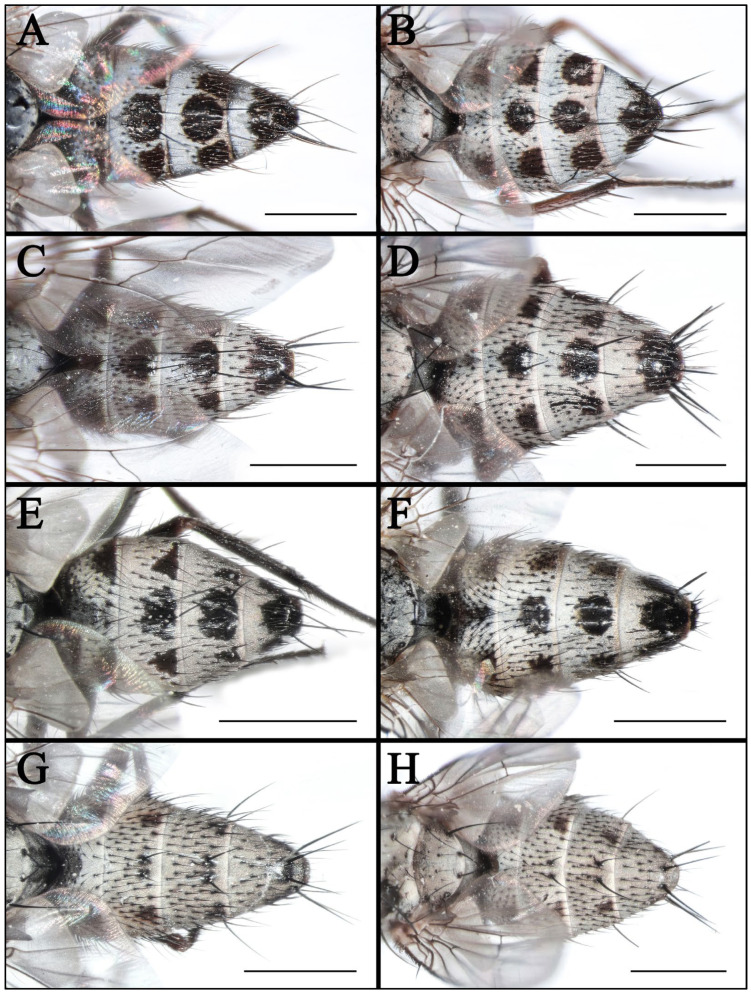
Abdomina of *Taxigramma* spp., dorsal view. (**A**) *T. elegantula*, male; (**B**) *T. elegantula*, female; (**C**) *T. multipunctata*, male; (**D**) *T. multipunctata*, female; (**E**) *T. pluriseta*, male; (**F**) *T. pluriseta*, female; (**G**) *T. stictica*, male; (**H**) *T. stictica*, female. Scale bars: (**A**–**H**) 1 mm.

**Figure 5:**
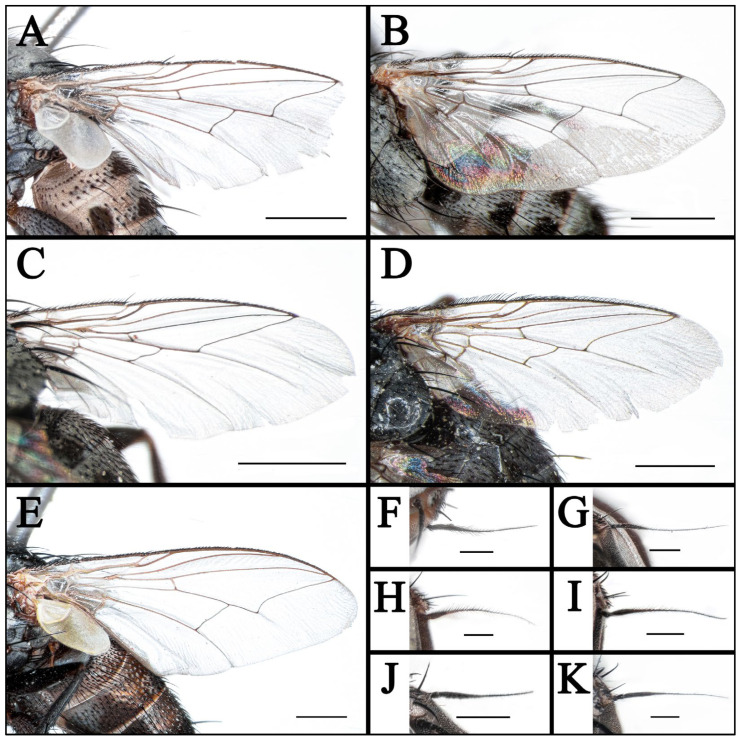
(**A**–**E**) Wings of *Taxigramma* spp.; (**F**–**K**) Aristae of *Taxigramma* spp., lateral view. (**A**) *T. albina*, female; (**B**) *T. albina*, male; (**C**) *T. heteroneura*, male; (**D**) *T. pluton*, male; (**E**) *T. pilicornis*, male; (**F**) *T. albina*, female; (**G**) *T. albina*, male; (**H**) *T. pilicornis*, male; (**I**) *T. stictica*, male; (**J**) *T. pluriseta*, male; (**K**) *T. pluton*, male. Scale bars: (**A**–**E**) 1 mm; (**F**–**K**) 0.2 mm.

**Figure 6:**
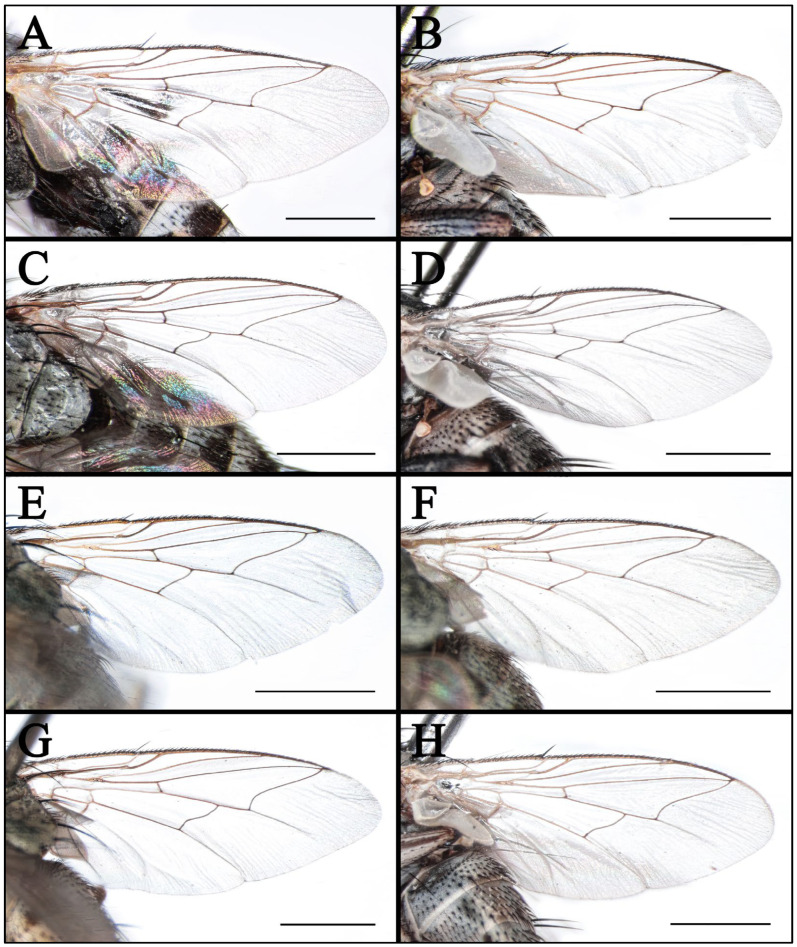
Wings of *Taxigramma* spp. (**A**) *T. elegantula*, male; (**B**) *T. elegantula*, female; (**C**) *T. multipunctata*, male; (**D**) *T. multipunctata*, female; (**E**) *T. pluriseta*, male; (**F**) *T. pluriseta*, female; (**G**) *T. stictica*, male; (**H**) *T. stictica,* female. Scale bars: (**A**–**H**) 1 mm.

**Figure 7:**
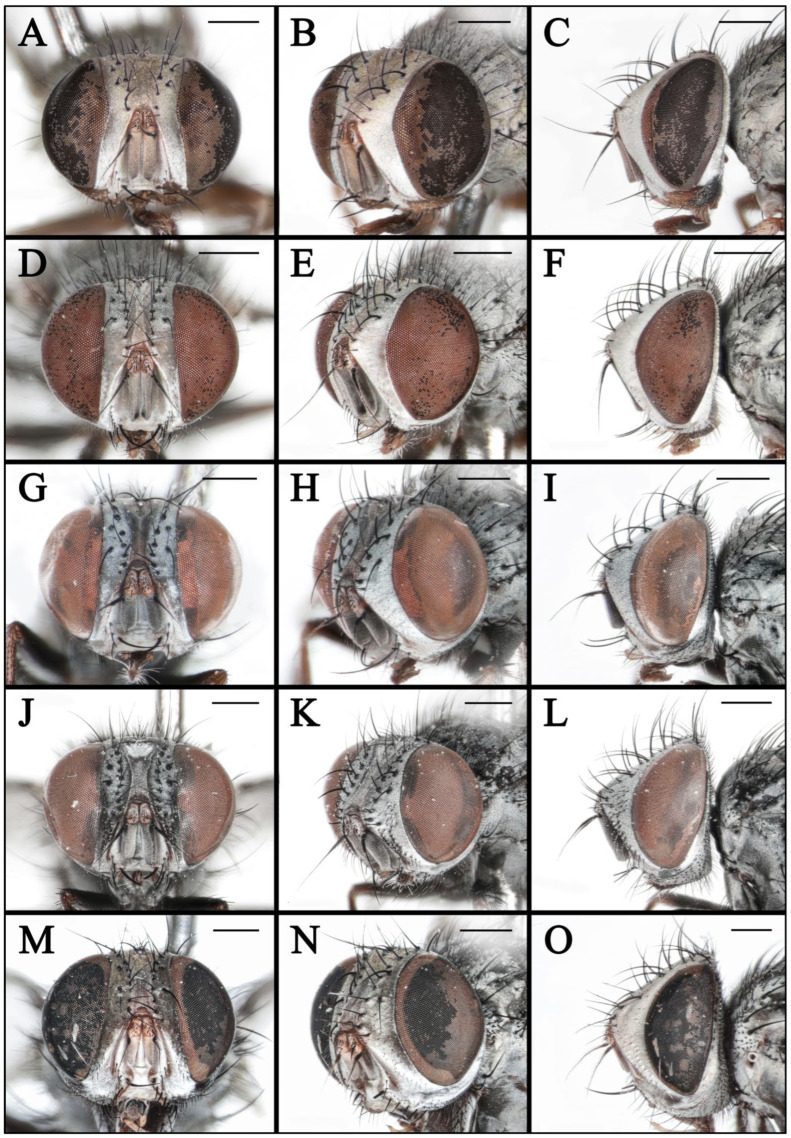
Heads of *Taxigramma* spp. (**A**–**C**) *T. albina*, female; (**D**–**F**) *T. albina*, male; (**G**–**I**) *T. heteroneura*, male; (**J**–**L**) *T. pluton*, male; (**M**–**O**) *T. pilicornis*, male. (**A**,**D**,**G**,**J,M**) anterior view; (**B**,**E**,**H**,**K,N**) anterolateral view; (**C**,**F**,**I**,**L**,**O**) lateral view. Scale bars: (**A**–**O**) 0.5 mm.

**Figure 8:**
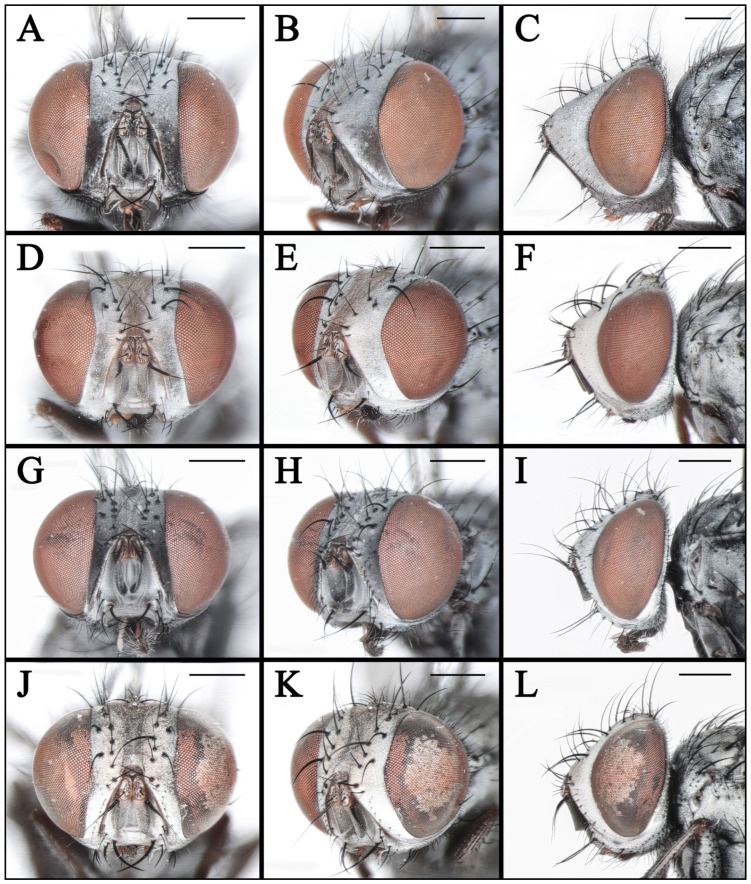
Heads of *Taxigramma* spp. (**A**–**C**) *T. elegantula*, male; (**D**–**F**) *T. elegantula*, female; (**G**–**I**) *T. multipunctata*, male; (**J**–**L**) *T. multipunctata*, female. (**A**,**D**,**G**,**J**) anterior view; (**B**,**E**,**H**,**K**) anterolateral view; (**C**,**F**,**I**,**L**) lateral view. Scale bars: (**A**–**L**) 0.5 mm.

**Figure 9:**
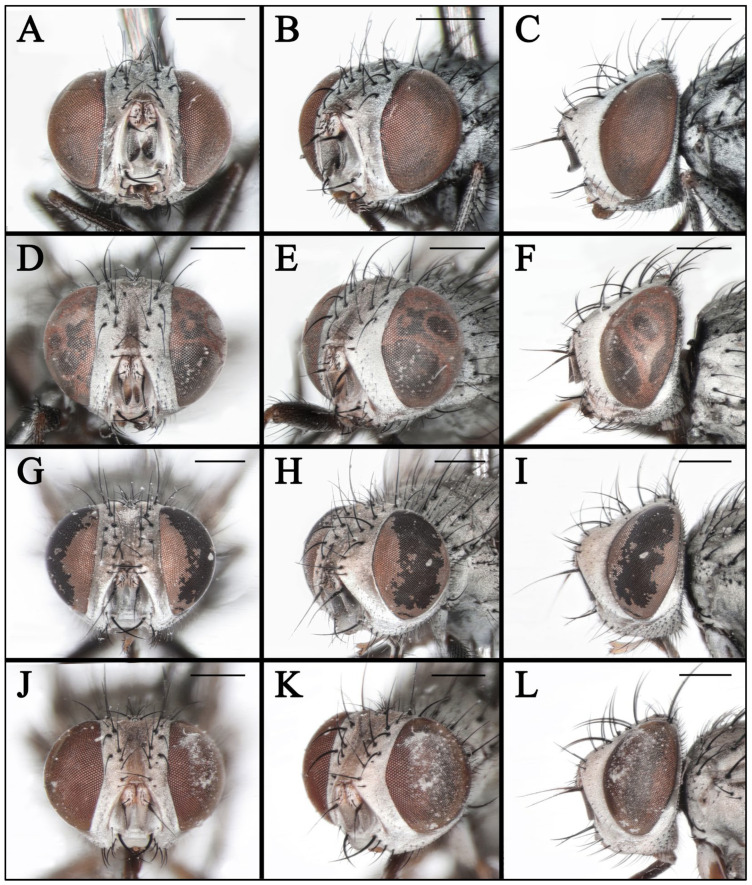
Heads of *Taxigramma* spp. (**A**–**C**) *T. pluriseta*, male; (**D**–**F**) *T. pluriseta*, female; (**G**–**I**) *T. stictica*, male; (**J**–**L**) *T. stictica*, female. (**A**,**D**,**G**,**J**) anterior view; (**B**,**E**,**H**,**K**) anterolateral view; (**C**,**F**,**I**,**L**) lateral view. Scale bars: (**A**–**L**) 0.5 mm.

1. Arista with medium-long plumosity in basal 1/2 (Figure 5H); proepisternal depression setose (Figure 1F); cell r_4+5_ narrowly open (Figure 5E) ………***T. pilicornis***
– Arista with short plumosity or pubescence; proepisternal depression bare; cell r_4+5_ closed at wing margin or petiolate ………………………………………………………………………2
2. Arista with short plumosity (Figure 5F,G,I); the distal section of the vein CuA_1_ distinctly shorter than its preceding section (Figure 5A,B and Figure 6G,H) ………………………3
– Arista almost bare, only with pubescence (Figure 5J,K); the distal section of the vein CuA_1_ typically equal to or longer than its preceding section (Figure 5C,D and Figure 6A–F) ………………………………………………………………………………………………………………………4
3. Abdomen without large, complete median spots, tergite 3 to tergite 5 each with one pair of small spots at the base of the median marginal bristles; body densely covered with yellowish-gray pollinosity (Figure 2G,H and Figure 4G,H); male fore tarsus with about 8 fine, hair-like setae on the posterior surface (Figure 3I) …………***T. stictica***
– Abdomen with large, complete median spots; body densely covered with light silvery gray pollinosity (Figure 1A,B and Figure 3A,B); male fore tarsomeres 1–5 each with a pair of fine, hair-like setae apically (Figure 3F) …………………………………***T. albina***
4. Male claws and pulvilli large, the former approximately equal in length to tarsomere 5, while the latter distinctly longer than tarsomere 5 (Figure 3J); distal section of vein CuA_1_ more than 2.00 × as long as the preceding section (Figure 5C); male terminalia prominently protruding, extending beyond tergite 5 ……………………***T. heteroneura***
– Male claws and pulvilli small, both shorter than tarsomere 5; distal section of vein CuA_1_ at most 1.50 × as long as the preceding section (Figure 5D and Figure 6A–F); male terminalia non-protruding ………………………………………………………………………5
5. The length of the post-bend section of vein M approximately equal to the length between the bend of vein M and crossvein r-m; cell r_4+5_ closed at wing margin or petiolate (Figure 6A,B,E,F); male fore tarsus with about 8 fine, hair-like setae (Figure 3G,H); the frons wide in lateral view, protruding anteriorly (Figure 8C and Figure 9C) ………………………………………………………………………………………………………………………6
– The length of the post-bend section of vein M about 1.25–1.50 × as long as the length between the bend of vein M and crossvein r-m; cell r_4+5_ closed at wing margin (Figure 5D and Figure 6C,D); male fore tarsus without fine, hair-like setae; the frons narrow in lateral view, non-protruding anteriorly (Figure 7L and Figure 8I,L) ………………………………………………………………………………………………………………………7
6. Head with dense light silvery gray pollinosity; gena tapering gradually downward; vibrissal angle non-protruding (Figure 8C,F); proboscis short, not reach the anterior margin of the eye in lateral view; cell r_4+5_ closed at wing margin or indistinctly petiolate (Figure 6A,B) ………………………………………………………………………***T. elegantula***
– Head with sparse silvery gray pollinosity, revealing the yellowish ground coloration; gena with nearly parallel inner and outer margins; vibrissal angle protruding anteriorly (Figure 9C,F); proboscis long, distinctly extends beyond the anterior margin of the eye in lateral view; cell r_4+5_ distinctly petiolate (Figure 6E,F) …………………………………………………………………………………***T. pluriseta***
7. Abdomen without large, complete median spots, tergite 3 to tergite 5 each with one pair of small spots at the base of the median marginal bristles, indistinctly fused into a vestigial median spot (Figure 3D); the length of the post-bend section of vein M about 1.25 × as long as the length between the bend of vein M and crossvein r-m; wing margin with long, distally recurved bristles arranged in a uniformly spaced, pectinate series (Figure 5D); body generally dark-colored (Figure 1D) ……………***T. pluton***
– Abdomen with large, complete median spots on tergite 3 to tergite 5 (Figure 4C,D); the length of the post-bend section of vein M about 1.50 × as long as the length between the bend of vein M and crossvein r-m; wing margin with short, straight setae arranged irregularly (Figure 6C,D); body generally light-colored (Figure 2C,D) ………………………………………………………………………***T. multipunctata***

**Figure 10:**
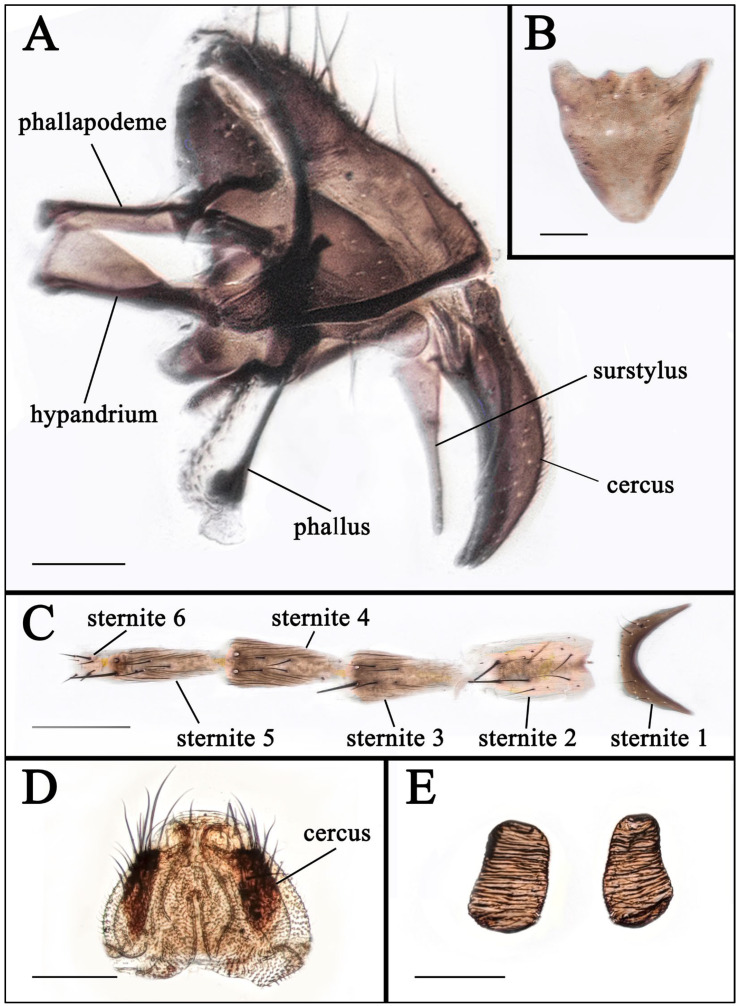
Terminalia of *T. albina*. (**A**) Terminalia, male, lateral view. (**B**) Sternite 5, male, ventral view. (**C**) Sternites 1−6, female, ventral view. (**D**) Terminalia, female, ventral view. (**E**) Spermathecae. Scale bars: (**A**,**B**,**D**,**E**) 0.1 mm; (**C**) 0.4 mm.

### 3.3. Genetic Distance Analysis

A total of 20 COI sequences were successfully obtained, representing the following species: *T. elegantula* (n = 5), *T. multipunctata* (n = 4), *T. heteroneura* (n = 3), *T. hilarella* (n = 2), *T. stictica* (n = 2), *T. albina* (n = 1), *T. pluton* (n = 1), *T. pluriseta* (n = 1) and *T. pilicornis* (n = 1).

Analysis of pairwise K2P genetic distances based on COI sequences among *Taxigramma* species is presented in Table 3. Maximum intraspecific K2P distances within *Taxigramma* were ≤2.94%, falling below the standard animal COI barcoding threshold of 3%. Species-level maximum intraspecific distances were: *T. elegantula* (2.94%), *T. multipunctata* (1.04%), *T. heteroneura* (0.77%), *T. hilarella* (0.34%) and *T. stictica* (0%). We found no genetic divergence (K2P = 0%) between the Sichuan (ecotone) and Hainan (Oriental) populations of *T. multipunctata*. In contrast, both of these populations from southern China exhibited a genetic distance of 0.78% to the population from Xinjiang (Palearctic).

Minimum interspecific K2P distance was 3.75% (between *T. elegantula* and *T. hilarella*), while the maximum was 15.10% (between *T. pilicornis* and *T. stictica*). A distinct barcoding gap of 0.81% was observed, with no overlap between intra- and interspecific genetic distances. These results strongly support the utility of the COI gene for species identification within *Taxigramma*.

**Table 3:** Pairwise K2P genetic distances (%) based on COI sequences among *Taxigramma* species.

		1	2	3	4	5	6	7	8	9	10	11	12	13	14	15	16	17	18	19	20
1	*T. albina* (PX133180)																				
2	*T. elegantula* (KY749760)	6.16																			
3	*T. elegantula* (MZ623189)	5.83	0.17																		
4	*T. elegantula* (MZ623596)	5.66	0.34	0.31																	
5	*T. elegantula* (MZ627095)	5.30	1.02	0.92	0.92																
6	*T. elegantula* (PX133185)	6.44	2.28	2.94	2.52	1.67															
7	*T. heteroneura* (KY749761)	11.33	11.64	11.44	11.66	11.04	10.80														
8	*T. heteroneura* (MN411060)	10.62	11.64	10.95	10.96	10.42	8.72	0.00													
9	*T. heteroneura* (PX133184)	12.29	13.42	13.19	13.19	12.96	14.97	0.77	0.77												
10	*T. hilarella* (KY749762)	5.58	4.53	4.35	4.53	3.99	4.61	10.27	10.27	12.05											
11	*T. hilarella* (MZ628314)	5.14	4.17	4.23	4.24	3.75	4.23	10.08	9.38	11.83	0.34										
12	*T. multipunctata* (KY749763)	10.69	10.48	10.28	10.49	9.89	10.16	6.33	6.33	7.25	8.74	8.55									
13	*T. multipunctata* (PX133186)	7.75	9.32	9.05	8.62	8.62	9.18	7.04	7.33	8.91	7.59	6.90	1.04								
14	*T. multipunctata* (PX133187)	6.90	8.74	8.18	7.75	7.75	8.26	6.46	6.48	8.18	7.03	6.06	0.52	0.78							
15	*T. multipunctata* (PX133188)	6.90	8.74	8.18	7.75	7.75	8.26	6.46	6.48	8.18	7.03	6.06	0.52	0.78	0.00						
16	*T. pilicornis* (KY749727)	13.10	11.87	11.66	11.88	11.85	13.49	13.06	13.06	14.61	12.49	12.29	12.26	12.96	13.59	13.59					
17	*T. pluriseta* (PX133182)	11.96	14.71	13.37	13.17	13.17	13.50	12.90	12.38	14.04	14.26	13.38	10.67	10.83	10.39	10.39	13.59				
18	*T. pluton* (PX133181)	8.88	9.50	9.10	8.88	8.65	9.16	8.51	8.23	9.09	10.00	8.88	7.03	7.26	6.37	6.37	13.42	11.61			
19	*T. stictica* (KY749764)	8.61	8.00	7.81	8.01	7.43	12.76	11.24	11.24	12.96	6.69	6.32	9.89	10.50	9.90	9.90	13.88	13.80	10.00		
20	*T. stictica* (PX133183)	8.11	8.92	8.65	8.47	8.09	11.54	12.57	11.71	13.65	7.45	6.80	11.04	10.38	9.49	9.49	15.10	12.57	9.33	0.00	

**Figure 11:**
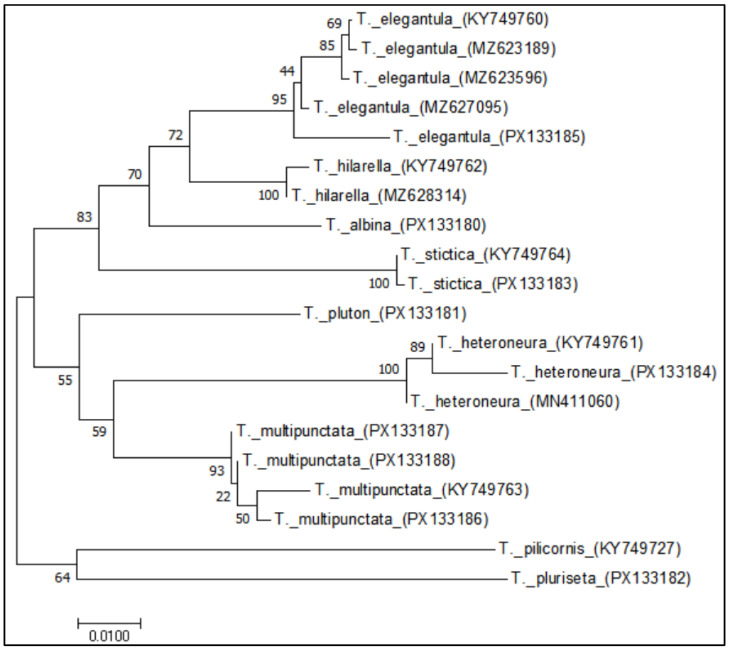
NJ tree of *Taxigramma* species based on the COI gene.

Furthermore, the NJ tree based on COI sequences demonstrates robust support for the monophyly of *T. multipunctata*, *T. elegantula*, *T. heteroneura*, *T. hilarella* and *T. stictica*, with bootstrap values of 93%, 95%, 100%, 100% and 100% respectively (see Figure 11). However, significant length heterogeneity among COI sequences may introduce sampling error in genetic distance estimation for short-sequence specimens, reduced branch support metrics.

Collectively, the combination of low intraspecific genetic distances (ranging from 0% to 2.94% in the species studied) and the highly supported monophyly of all species in the NJ tree strongly supports the utility of the COI gene as an effective molecular marker for species identification within the *Taxigramma*. Although observed length heterogeneity in some sequences may introduce sampling error, it does not appear to compromise the overall resolution at the species level, as evidenced by the high bootstrap values.

## 4. Discussion

In taxonomic studies of the genus *Taxigramma*, easily observable features of the external morphology including details of head, wing, chaetotaxy and abdomen (Figure 1, Figure 2, Figure 3, Figure 4, Figure 5, Figure 6, Figure 7, Figure 8 and Figure 9) as well as more complex details of the terminalia (Figure 10) are treated as critical diagnostic characteristics [5,6,8,16,17,18,26,27]. Pape [1] and Szpila [28] also noted that the proepisternal depression of *T. pilicornis* is setose, which not only serves as a reliable diagnostic character for distinguishing it from other *Taxigramma* species but also represents an exceptionally rare morphological characteristic within the Sarcophagidae. We additionally observed that certain species [e.g., *T. albina*, *T. elegantula*, *T. pluriseta* and *T. stictica*] possess distinctive hair-like setal structures on male fore tarsus, which appear to have diagnostic value for taxonomic identification.

Several researchers have recently conducted a series of studies on *Taxigramma* employing molecular data [2,14,15] and the morphology of first-instar larvae [29], leading to both refinement of its taxonomic system and proposal of novel diagnostic characteristics. Our research provides additional molecular data, and based on genetic distance analysis, we demonstrate the critical role of COI sequences in species identification (Figure 11), further highlighting the significance of integrating morphological characteristics with molecular evidence in taxonomic research.

The vast majority of *Taxigramma* species exhibit a Palaearctic–Afrotropical distribution pattern, with only a few species extending into the Nearctic and Oriental regions [1,3,4,5,6,7,8,16,18,26,27,30,31,32,33,34,35,36]. In China, most species of *Taxigramma* are primarily restricted to deserts, grassland and Gobi areas in the northern and northwestern regions [1,3,5,6,7,8]. Notably, this study provides the first record of *T. multipunctata* within the Oriental part of China in Hainan and Yunnan provinces. An analysis of intraspecific genetic distances from specimens collected in Xinjiang, Sichuan, and Yunnan showed no significant differences. Further analysis should be carried out by obtaining longer sequences.

**Figure 12:**
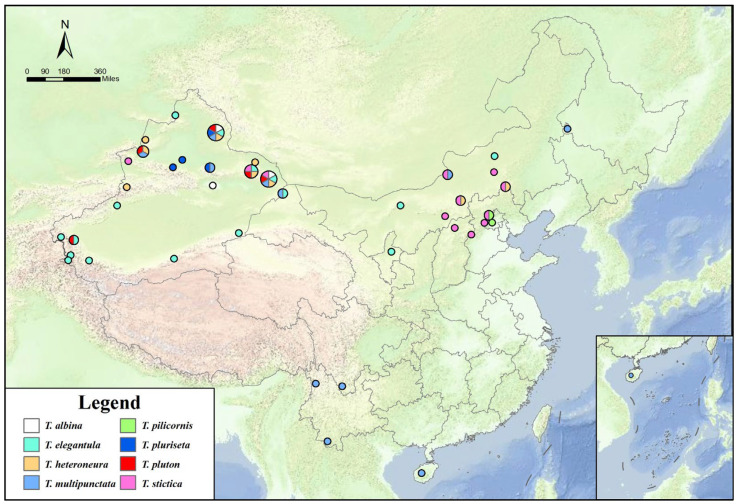
Geographical distribution of *Taxigramma* species from China.

All new country records reported by the present research are from Xinjiang, which is one of the regions exhibiting the highest diversity of Miltogramminae in China [8]. Among these, Turpan-Hami Basin and Kalamaili are particularly representative. Their typical desert and semi-desert habitats make this region a critical site for research on the subfamily Miltogramminae (Figure 12).

## 5. Conclusions

This study provides the first review of the genus *Taxigramma* from China, including an updated identification key with the two newly recorded species, *T. pluriseta* and *T. pluton*. Detailed information is given on the distribution in China for all eight species of *Taxigramma*, including the first record of *T. multipunctata* from the Oriental part. Examined specimens are listed and the first description of the male *T. albina* is provided as well as the first COI sequences for *T. multipunctata*, *T. pluriseta* and *T. pluton*. The species *T. karakulensis* could not be confirmed from China and is omitted from the checklist.

## Figures and Tables

**Table 1:** Primers used in this study.

Primer	Forward/Reverse	Sequence
LCO1490	Forward	5′-GGTCAACAAATCATAAAGATATTGG-3′
HCO2198	Reverse	5′-TAAACTTCAGGGTGACCAAAAAATCA-3′
miniBarcode-F	Forward	5′-GGWACWGGWTGAACWGTWTAYCCYCC-3′
miniBarcode-R	Reverse	5′-TANACYTCNGGRTGNCCRAARAAYCA-3′

## Data Availability

All data produced during this research are included in this publication and can be obtained by contacting the corresponding author.

## Abbreviations

The following abbreviations are used in this manuscript:

BFU	Beijing Forestry University, Beijing, China
IZCAS	Institute of Zoology, Chinese Academy of Sciences, Beijing, China
SYNU	Shenyang Normal University, Shenyang, Liaoning, China
